# Subsequent Device Usage and Caregiver Attitudes to Do-It-Yourself Real-Time Continuous Glucose Monitoring (DIY-rtCGM) among Children with Type 1 Diabetes 3 Months after Participation in a Randomized Controlled Trial

**DOI:** 10.1155/2023/3435944

**Published:** 2023-08-25

**Authors:** Yongwen Zhou, Mona M. Elbalshy, Sara E. Styles, Hamish Crocket, Craig Jefferies, Esko Wiltshire, Martin I. de Bock, Benjamin J. Wheeler

**Affiliations:** ^1^Department of Women's and Children's Health, Dunedin School of Medicine, University of Otago, Dunedin, New Zealand; ^2^Department of Endocrinology, Institute of Endocrine and Metabolic Diseases, The First Affiliated Hospital of USTC, Division of Life Sciences and Medicine, Clinical Research Hospital of Chinese Academy of Sciences (Hefei), University of Science and Technology of China, Hefei, Anhui 230001, China; ^3^Department of Human Nutrition, University of Otago, Dunedin, New Zealand; ^4^Te Huataki Waiora School of Health, University of Waikato, Hamilton, New Zealand; ^5^Starship Child Health, Te Whatu Ora–Health New Zealand, Te Toka Tumai Auckland, New Zealand; ^6^Liggins Institute, University of Auckland, Auckland, New Zealand; ^7^Department of Paediatrics and Child Health, University of Otago Wellington, Wellington, New Zealand; ^8^Paediatrics and Child Health, Te Whatu Ora/Health New Zealand, Wellington, New Zealand; ^9^Department of Paediatrics, University of Otago Christchurch, Christchurch, New Zealand; ^10^Department of Paediatrics, Te Whatu Ora-Waitaha, Christchurch, New Zealand; ^11^Paediatric Endocrinology, Te Whatu Ora/Health New Zealand-Southern, Dunedin, New Zealand

## Abstract

**Aim:**

To assess children's subsequent device usage and caregiver attitudes to do-it-yourself real-time continuous glucose monitoring (DIY-rtCGM) at least 3 months after completing a randomized controlled trial (RCT).

**Methods:**

A brief online questionnaire or telephone call was used to collect the subsequent device usage and caregivers' attitudes from a total of 55 families at least 3 months after their completion of an RCT investigating DIY-rtCGM adapted from their preexisting intermittently scanned glucose sensors plus education on using DIY-rtCGM system. To be eligible for the RCT, children had to be aged 2–13 years, have type 1 diabetes ≥6 months, and be rtCGM naïve. Data collected investigated current CGM use post-RCT and attitudes/user experiences to DIY-rtCGM in the months since RCT study support ended.

**Results:**

Overall, responses from 81.8% (45/55) of caregivers were received. Mean age of children was 9.0 ± 2.7 years, and 31 (68.9%) children used insulin pumps. After 3 months, 44.4% (20/45) of responding caregivers reported ongoing DIY-rtCGM use, and of these, only 13 used DIY-rtCGM as the primary glucose monitoring method 100% of time. Of the 25 (55.6%) families who ceased DIY-rtCGM, 40% (10/25) had transitioned to commercial rtCGM. More than half of families (60%, 12/20) who continued DIY-rtCGM use had a very or extremely positive attitude toward the technology and 75% (15/20) of these families planned to continue DIY-rtCGM use. However, signal loss and sensor inaccuracy remained the major reasons among all responders both for decreased DIY-rtCGM wear time and eventual cessation. Burden of use primarily related to technical errors that could not be solved, and alarms, both of which were reported to contribute to discontinuation.

**Conclusions:**

This study highlights that, among families voluntarily using DIY-rtCGM at least 3 months following support from a trial, more than half have ceased using DIY-rtCGM, with 40% of those discontinuing switching to commercial rtCGM. While overall perceptions of DIY-rtCGM remain largely positive, burdens of use are high and contribute to discontinuation.

## 1. Introduction

The role of continuous glucose monitoring (CGM) in the management of type 1 diabetes (T1D) has become increasingly important, with CGM now recommended in all major guidelines [1–3]. Both forms of CGM, real-time CGM (rtCGM) and intermittently scanned CGM (isCGM), have shown a range of benefits [1, 4].

However, evidence for rtCGM appears to show advantages over first-generation isCGM with regard to glucose control [5–9] (no comparative data available for second-generation isCGM). In addition, rtCGM also offers real-time glucose data, glucose threshold alerts (limited alerts are available in second-generation isCGM), and the potentials for remote monitoring by family or caregivers [10]. However, full function rtCGM generally incurs almost double the cost of first- and second-generation isCGM; although since conducting this study, lower cost rtCGM is starting to emerge. Cost has, therefore, contributed to inequity in access to diabetes technology and lack of funding in many regions worldwide remains a major issue [11–13].

Driven by the “#WeAreNotWaiting” innovation and advocacy movement, do-it-yourself rtCGM (DIY-rtCGM) emerged as an alternative cheaper option for rtCGM. This is done by adapting the isCGM sensor with a third-party Bluetooth transmitter and smartphone app. DIY-rtCGM offers many of the advantages of commercial rtCGMs yet at a potential reduced cost (isCGM costs plus one-off upfront cost of a reusable Bluetooth transmitter, usually <$200 USD). It has been adopted by some worldwide [14] but specific numerical data on uptake remain unknown. Recently, we reported positive randomized controlled trial (RCT) data in children highlighting that DIY-rtCGM appears to offer additional glucose control and diabetes treatment satisfaction benefits over first-generation isCGM [7]. However, there were still a variety of technical barriers reported, especially during the setup process and with Bluetooth communication [14].

This raises an interesting question of whether in an increasingly evolving CGM commercial landscape and outside of a supported research environment, first-time users of DIY-rtCGM would continue to find benefits or transition to alternative commercial rtCGM. Therefore, after the completion of MiaoMiao study [7], we assessed subsequent device usage and caregivers' attitudes to DIY-rtCGM use at 3 months posttrial.

## 2. Methods

We conducted a cross-sectional survey to follow up on DIY-rtCGM use and real-life experiences after finishing the MiaoMiao study [7, 14]. DIY-rtCGM system consisting of isCGM (FreeStyle Libre, Abbott Diabetes Care) and a Bluetooth transmitter (MiaoMiao version 2, Smart Reader, Shanghai High Brilliant Health Technology Co., Ltd., China). The Northern Health and Disability Ethics Committee approved this substudy (19/NTB/118/AM03).

### 2.1. Procedures

Caregivers (*n* = 55) of children aged between 2 and 13 years with type 1 diabetes for a minimum of 6 months and rtCGM naïve at the start of MiaoMiao study were invited to complete a brief online or phone questionnaire (for nonresponders to online) reporting on their experiences in the 3-month period after trial completion. All responders gave their consent. Up to two email reminders were sent to nonresponders, and if still not responded, telephone calls were subsequently made for retrospective report on this original 3-month time period. Data were recorded and managed using secure Research Electronic Data Capture tools hosted at Otago University.

### 2.2. Measures

The questionnaire used logic branching to explore user experiences according to participants' subsequent use of DIY-rtCGM using the question: “Does your child still use MiaoMiao for continuous glucose monitoring?” (full questionnaire details in supplementary document). In addition to the question items, free-text options were available for caregivers to elaborate on their experiences.

### 2.3. Analysis

For quantitative data, descriptive statistics were calculated as means and standard deviations, or counts and proportions as appropriate, to describe respondent characteristics and current practices. Two-sample *t*-test for continuous variables and a *χ*^2^ test for categorical variables were used to examine the differences in demographics between families with ongoing use and those who had ceased. Open-ended responses were also analyzed by two independent reviewers to arrive at major themes. Statistical analyses were conducted using R (3.5.2).

## 3. Results

### 3.1. Respondent Demographics

The response rate was 81.8% (45/55), with 29 finishing the online questionnaire and 16 subsequently reporting via phone calls. Demographic information is provided in Table 1. The mean age of children is 9.0 ± 2.7 years, with 25 (55.6%) being female and 31 (68.9%) using insulin pumps at RCT baseline.

### 3.2. Ongoing Use of DIY-rtCGM Posttrial Support

Twenty (44.4%) caregivers reported their family were still using DIY-rtCGM at least occasionally. Of these, only 65% (13/20) used DIY-rtCGM as the primary glucose monitoring method for 100% of the time. All 20 caregivers acknowledged that they continued to find DIY-rtCGM beneficial with 60% (12/20) reporting it as very or extremely beneficial. In addition, 75% (15/20) planned continued use of DIY-rtCGM. No respondents reported that they had purchased an additional MiaoMiao Bluetooth transmitter device as a backup device.

There were 25 families (55.6%, 25/45) who had ceased DIY-rtCGM within 3 months following the RCT end. Among these families, 40.0% (10/25) chose to self-fund commercial rt-CGM. Of those where data were available, commercial systems reverted to included: Dexcom G6 (*n* = 9), Medtronic Guardian (*n* = 1), and 14 participants reverted back to their original first-generation isCGM (Abbott Freestyle Libre).

No significant demographic differences were seen between families/children with ongoing use and those who had discontinued (all *P* > 0.05).

### 3.3. Perceived Barriers to Ongoing Posttrial DIY-rtCGM Use

Among 29 families who provided perceived barriers to ongoing use, 35% (10/29) of responders reported they had experienced technical issues with frequency of < once/week (*n* = 3), 1–2 times/week (*n* = 3), 6–7 times/week (*n* = 2), and > once/day on average (*n* = 2). Signal loss (via Wi-Fi/Bluetooth) requiring a reconnection was the most common technical issue (*n* = 8). Another problem noted was inconvenience uploading data for diabetes healthcare visits (*n* = 1). Three noted more complex problems, which were time-consuming to fix, including cache memory issues (*n* = 1), variable glucose recordings requiring additional calibration (*n* = 1), and inability to calibrate (*n* = 1).

Technical issues such as signal loss (*n* = 4) and glucose inaccuracy (*n* = 1) were given as reasons for not always wearing DIY-rtCGM. Alarm fatigue, including connectivity alarms (*n* = 1) and annoyance at carrying the charging equipment (*n* = 1), were other issues reported.

### 3.4. Reasons for DIY-rtCGM Discontinuation

Fourteen families shared their reasons for discontinuation. More than half (9/14, 64.3%) reported problems with either the follower or collector phone were one of the major reasons for their cessation. Concerns regarding glucose reporting accuracy (5/14, 35.7%) and unresolvable DIY-rtCGM technical issues (11/14, 78.6%) were the other reasons contributing to cessation. The burden of keeping DIY-rtCGM working was “just too much” for 5/14, two responders reported there were too many connectivity alarms, and two reported their children disliked wearing the device.

## 4. Discussion

This study reports the results of a posttrial assessment of subsequent usage and caregiver attitudes and experiences to DIY-rtCGM. This is important, as to date DIY technology use has been largely driven by enthusiastic and skilled early adopters as opposed to those reported here—first-time users of DIY-rtCGM as part of a trial. As shown, less than half of responding caregivers continued to use DIY-rtCGM, with many of those discontinuing transferring to commercial rtCGM. Technical challenges remained an ongoing issue, including concerns with signal loss/connectivity alarms and perceived sensor accuracy.

For these first-time users of DIY-rtCGM, burden related to technical problems remained a major reason for negative experience and eventual discontinuation. This differs somewhat from the recently reported generally positive experiences of ongoing users and early self-adopters of DIY-rtCGM [14]. Although early adopting caregivers have noted complexity with the setup process, connectivity issues, and lack of support from medical teams when using DIY-rtCGM, overall perceptions of DIY-rtCGM remained largely positive with more than half planning for ongoing use and all stating they would recommend to another family [14]. DIY technology clearly differs from commercial use in that customer helplines are not available, support is largely online from DIY peers [15], and “invisible” interoperability barriers exist between the various component parts. All of these issues highlight important differences in potential experiences seen between skilled early adopters and those previously naïve to DIY diabetes technology. Diabetes teams and people with diabetes contemplating DIY-rtCGM use need to be cognizant of this potential discrepancy in reported experiences.

This rate of abandoning DIY-rtCGM by 3 months posttrial is also interesting given that in the primary trial, DIY-rtCGM showed similar clinically important glycemic benefits [7] to those seen in other rtCGM versus first-generation isCGM clinical trials [5–9]. One potential explanation for this is all in the original trials were naïve to rtCGM use, and, therefore, were not aware of the potential added benefits of rt-CGM compared to isCGM. This is highlighted in the fact that some of those reporting cessation of DIY-rtCGM had transitioned over to a commercial source for ongoing rtCGM. The need for DIY-rtCGM may largely be resolved as technology improves and cost of rtCGM lowers. Indeed, the first-generation isCGM has become obsolete in many countries, including New Zealand. The key point being those wishing to use rtCGM should ideally have access to it, as per all recent guidelines [1–3].

A key strength of this study is the longitudinal assessment of DIY-rtCGM use posttrial, as opposed to just the experiences and outcomes noted during full study support. This adds a new dimension to the currently reported experiences of DIY-tech users, which have often focused on enthusiastic and skilled adopters. This study has limitations, including limitations in generalizability given the participants of predominantly European ethnicity and overall more privileged socioeconomic position; the potential for recall bias; and while data on continuation were available for >80%, a response bias may be an issue, with those nonresponding potentially overrepresented in discontinuation rates.

## 5. Conclusion

Despite glycemic benefits seen from the original RCT, less than half of these first-time users continued to use DIY-rtCGM at 3 months after the trial completion, with many who ceased use transitioning to traditional commercial rtCGM. Technical challenges remain a burden resulting in negative experience and eventual discontinuation of this DIY technology. Ongoing efforts to increase access and improve useability and burden of traditional rtCGM are required globally, which will minimize the need and the reported limitations of DIY-rtCGM.

## Figures and Tables

**Table 1:** Participant demographics.

Responding child–parent dyads	Total (*N* = 45)	Still using (*n* = 20)	Ceased use (*n* = 25)
Parent/caregiver			
Age (years), mean (SD)	40.47 (6.49)	41.60 (6.41)	39.56 (6.54)
Female, *n* (%)	44 (97.8)	20 (100.0)	24 (96.0)
Ethnicity, *n* (%)			
European	38 (84.4)	20 (100.0)	18 (72.0)
Maori	1 (2.2)	0 (0.0)	1 (4.0)
Other ^*∗*^	6 (13.3)	0 (0.0)	6 (24.0)
NZ deprivation index^†^, *n* (%)			
Low (1–3)	23 (51.2)	9 (45.0)	14 (56.0)
Medium (4–7)	15 (33.3)	8 (40.0)	7 (28.0)
High (8–10)	7 (15.5)	3 (15.0)	4 (16.0)
Full-time employment, *n* (%)	23 (52.3)	10 (50.0)	13 (54.1)
Marital status, *n* (%)			
Married	31 (68.9)	15 (75.0)	16 (64.0)
Partner/civil union	9 (20.0)	3 (15.0)	6 (24.0)
Separated/single	5 (11.1)	2 (10.0)	3 (12.0)
Child DIY-rtCGM user			
Age (years), mean (SD)	8.96 (2.66)	8.80 (2.31)	9.08 (2.96)
Female, *n* (%)	25 (55.6)	10 (50.0)	15 (60.0)
BMI *z*-score, mean (SD)	0.94 (1.74)	0.88 (1.82)	0.98 (1.71)
Diabetes duration (months), mean (SD)	36.81 (26.94)	36.85 (23.76)	36.78 (29.73)
Insulin therapy, *n* (%)			
CSII	31 (68.9)	15 (75.0)	16 (64.0)
MDI	14 (31.1)	5 (25.0)	9 (36.0)
HbA1c (%), mean (SD)	7.7 (3.1)	7.8 (2.4)	7.5 (2.5)
HbA1c (mmol/mol), mean (SD)	60.22 (10.61)	62.05 (6.48)	58.76 (12.97)

DIY-rtCGM, do-it-yourself real-time continuous glucose monitoring; MDI, multiple daily injection; CSII, continuous subcutaneous insulin injection; SD, standard deviation; BMI, body mass index; HbA1c, hemoglobin A1c.  ^*∗*^Other ethnicities: Samoan, *n* = 1; Chinese, *n* = 1; Others, *n* = 4. ^†^NZDep13 score is a deprivation index based on household address (where the participation lives more than 50% of the time) with 1 lease deprived and 10 most deprived.

## Data Availability

The data that support the findings of this study are available on request from the corresponding author. The data are not publicly available due to privacy or ethical restrictions.

## Supplementary Materials

Supplementary Materials Details on questionnaires of parents'/caregivers' experience after the MiaoMiao study.
