# Supplementary material for: Subsequent Device Usage and Caregiver Attitudes to Do-It-Yourself Real-Time Continuous Glucose Monitoring (DIY-rtCGM) among Children with Type 1 Diabetes 3 Months after Participation in a Randomized Controlled Trial
Source: Pediatr Diabetes. 2023 Aug 25;2023:3435944. doi: 10.1155/2023/3435944 (PMC12017063; doi:10.1155/2023/3435944)
Supplement: Supplementary Materials — Details on questionnaires of parents'/caregivers' experience after the MiaoMiao study. [file 3435944.f1.docx]

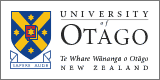


**Parents’/caregivers’ experience after the MiaoMiao study**

| **Date questionnaire completed** | Day | Month | Year |
| --- | --- | --- | --- |

This Survey contains questions about your experience after finishing the MiaoMiao study. This information is being collected to help us better describe the general characteristics of the study population.

This survey will take about 5 minutes to complete.

Please read each question carefully and answer questions honestly. All information you give is strictly confidential. You do not have to tell us anything that you do not feel comfortable sharing.

No one outside of the study research team will have access to this information.

**Thank you for participating in this Survey.**

1. **First name and surname of the person completing the questionnaire**

|  |
| --- |

1. **This questionnaire is being completed by (relationship to child in the Miaomiao study):**

☐ Mother

☐ Father

☐ Other (e.g. Grandma, please state

1. **Please confirm the following details:**

| **Street No/Name** |  |
| --- | --- |
| **Suburb** |  |
| **City** |  |
| **Landline** |  |
| **Cell phone** |  |
| **Email** |  |
| **MiaoMiao study ID** |  |

**Does your child still use MiaoMiao for Continuous glucose monitoring ?**

- Yes (Please go to part A) (even if intermittently)
- No (Please go to part B)

**Part A:**

**If your child is still using MiaoMiao, please answer the following questions:**

1. **What % of time in the past month have they used MiaoMiao with their Freestlye Libre? (you could even give % in 10% jumps)**

**If not used 100%, why not? (free text)**

1. **Please list the top 3 advantages of using MiaoMiao:**
2. ………………………………………………………………………………………
3. ………………………………………ad………………………………………………
4. ………………………………………………………………………………………
5. **Please list the top 3 disadvantages of using MiaoMiao:**
6. ………………………………………………………………………………………
7. ………………………………………………………………………………………
8. ………………………………………………………………………………………
9. **How often do you experience technical issues with MiaoMiao that need troubleshooting?**

**<once / week**

**1 x /week**

**2 x /week**

**3 x /week**

**4 x /week**

**5 x /week**

**6 x /week**

**7 x/week**

**>once / day on average**

- If you are experiencing technical issues that require some form of troubleshooting – please describe the nature and types experienced and you efforts to fix these.

1. **How beneficial has the MiaoMiao been for your child/ren?**

- Extremely beneficial.
- Very beneficial.
- Moderately beneficial.
- Slightly beneficial.
- Not beneficial at all.

1. **Have you bought another MiaoMiao as back up?**

- Yes
- No

1. **Are you planning to use MiaoMiao paired with FGM over the next 12 months?**

- Yes
- No

**Part B:**

**If your child is no longer using MiaoMiao, please answer the following questions:**

1. **When did your child stop using MiaoMiao? (approximate date)**

………………………………………………………………………………………

1. **Did you stop using MiaoMiao because of financial reasons?**

- Yes
- No

Please describe if needed

(use logic)

If no, please list the main reasons for stopping using MiaoMiao (free text)

1. ………………………………………………………………………………………
2. ………………………………………………………………………………………
3. ………………………………………………………………………………………
4. **Do you currently use another technology (device) for continuous glucose monitoring?**

- Yes
- No

If Yes, please tick the box/es below if you are using one or more of the following

- BluCon
- Dexcom G6
- Dexcom G5
- Medtronic Guardian
- Freestyle Libre
- Other

If not listed above please state the name/s of other device/s you are currently using.

………………………………………………………………………………………

………………………………………………………………………………………
